# Using narratives to correct politically charged health misinformation and address affective belief echoes

**DOI:** 10.1093/pubmed/fdae050

**Published:** 2024-04-18

**Authors:** Helen M Lillie, Chelsea L Ratcliff, Andy J King, Manusheela Pokharel, Jakob D Jensen

**Affiliations:** Department of Communication Studies, University of Iowa, Iowa City 52242, USA; Department of Communication Studies, University of Georgia, Athens 30602, USA; Department of Communication, University of Utah, Salt Lake City 84112, USA; Department of Communication Studies, Texas State University, San Marcos 78666, USA; Department of Communication, University of Utah, Salt Lake City 84112, USA

**Keywords:** COVID-19, misinformation, narrative, political ideology, relief

## Abstract

**Background:**

In May 2020, news outlets reported misinformation about the Centers for Disease Control (CDC) related to COVID-19. Correcting misinformation about outbreaks and politics is particularly challenging. Affective belief echoes continue to influence audiences even after successful correction. Narrative and emotional flow scholarship suggest that a narrative corrective with a positive ending could reduce belief echoes. Therefore, this study investigated the efficacy of a narrative corrective with a relief ending for correcting misinformation about the CDC.

**Methods:**

Between 29 May and 4 June 2020, we tested the effectiveness of a narrative to correct this misinformation. Participants in the United States (*N* = 469) were enrolled via Qualtrics panels in an online message experiment and randomized to receive a narrative corrective, a didactic corrective or no corrective.

**Results:**

The narrative corrective resulted in lower endorsement of the misinformation compared with the control and the didactic corrective. The narrative corrective had a positive indirect effect on perceived CDC competence and mask wearing intentions for politically moderate and conservative participants via relief.

**Conclusions:**

Public health institutions, such as the CDC, should consider utilizing narrative messaging with positive emotion endings to correct misinformation. Narratives better address affective belief echoes, particularly for counter-attitudinal audiences.

The COVID-19 pandemic highlighted the spread of health misinformation in the new media environment.^1^ Health misinformation proliferates on social media, often packaged in sensational, emotion-laden narratives.^2^ During a crisis, it is vital that public health officials engage in empirically supported communication strategies to reduce the public’s risks, including the risks posed by misinformation.^3^ While misinformation research often focuses on incorrect health information, misinformation and conspiracy theories about public health institutions also abound.^4^ Such stories can damage trust in institutions, such as the Centers for Disease Control (CDC), diminishing adherence to health guidelines.^5–7^ Indeed, trust in the competence of government and public health officials motivated prevention behaviors during the COVID-19 pandemic.^8–10^ Therefore, the primary goal of this study is to test messaging strategies for correcting misinformation about public health institutions, specifically the CDC.

Misinformation often continues to influence attitudes, behaviors and beliefs after receipt of corrective information.^11^^,^^12^ Misinformation has a ‘continued influence’ when the original misinformation is still believed to be factual despite correction.^13^^,^^14^ This can occur because misinformation fits in an existing mental model or aligns with a valued belief system, such as political ideology.^13^ Furthermore, ‘affective belief echoes’ arise when the misinformation influences attitudes and behaviors even though the misinformation is no longer believed to be factual.^15^ This occurs because the emotional residue of the misinformation lingers, creating a negative association with the object of the misinformation.

Scholars have debated if narratives are more effective than didactic messaging for correcting misinformation.^16^ A narrative is a ‘cohesive and coherent story with identifiable beginning, middle, and end that provides information about scene, characters, and conflict; raises unanswered questions or unresolved conflict; and provides resolution’ (p. 778).^17^ Narrative correctives should be effective at addressing the affective component of misinformation because narratives typically produce stronger emotional reactions than non-narratives.^18–20^ Indeed, research in the tobacco context has found that narrative correctives outperform non-narratives.^16^^,^^21^ However, another study found no difference between corrective types.^22^

This inconsistency could be because narrativity itself is not sufficient. Sangalang* et al.* argue that an explicitly emotional ending is needed for effective narrative correctives.^16^ Additionally, the context may matter. Misinformation about outbreaks and politics are more challenging to correct than other types of misinformation,^23^^,^^24^ possibly necessitating a narrative strategy. Ecker *et al*.’s work^22^—finding no difference between corrective types—covered a range of issues but only two of these (salmonella outbreak and mental health of children with homosexual parents) involved outbreaks or highly politicized information. Therefore, another goal of this study is to test if a narrative corrective with an emotional ending outperforms a didactic corrective in a highly politicized, outbreak context (the COVID-19 pandemic).

Prike and Ecker^25^ include narrativity as a factor that ‘might matter’ for corrective effectiveness, calling for more research assessing correction format and/or medium. Although belief echoes theorizing does not argue for the superiority of either message form,^15^^,^^25^ recent meta-analyses outside the corrective context have found narratives to be more persuasive.^26–28^ Therefore, we expect that a narrative corrective with an emotional ending, compared with both a didactic corrective and a no-correction control, should diminish belief in misinformation (H1), produce greater perceived CDC competence (H2) and produce greater mask wearing intention (H3).

Specifically, we test the inclusion of a positive emotion ending. Recent emotion and messaging scholarship has forwarded the concept of emotional flow—that messages typically produce multiple emotions in audiences that ebb and flow throughout the message.^29^ Research thus far supports a flow that begins with negative feeling (e.g. anxiety, frustration) and ends on a positive feeling (e.g. hope).^30–32^ Misinformation about the CDC could lead to frustration or worry because it implies the CDC cannot competently respond to public health crises.^33^ Therefore, an effective narrative should acknowledge those feelings and then transition to a positive ending, resulting in a state of relief. Relief is a positive emotion that arises from the removal or avoidance of a goal-incongruent, unpleasant or threatening situation.^34^^,^^35^ We expect that a narrative corrective with a positive emotional ending will indirectly affect belief in misinformation (H4), perceived CDC competence (H5) and mask wearing intention (H6) through feelings of relief.

Political misinformation can be more challenging to correct, and continued influence can occur when misinformation aligns with political ideology.^13^^,^^24^ COVID-19 risk perception and preventative behaviors, including mask wearing, varied with political ideology.^36^^,^^37^ Specifically, individuals with a conservative ideology were less likely to mask and had greater mistrust for public health organizations.^36^^,^^37^ To assess how the political context influences the effectiveness of corrective type, we ask if political ideology moderates the hypothesized relationships (RQ).

## Method

### Participants and procedures

Participants were recruited via Qualtrics Panels, with data collection occurring from 29 May 2020 to 4 June 2020. Qualtrics Panels recruits participants from their participant pool. Interested participants followed a link to the survey on the Qualtrics survey platform. All participants received a misinformation message about the CDC. Participants were then randomly assigned to one of three conditions (narrative corrective, didactic corrective or no-correction control). After reading the message, participants responded to measures about their emotional reaction, perceptions of the CDC, belief in the misinformation and mask wearing intentions. All participants were debriefed at the end of the survey. Qualtrics removed participants who rushed through the survey, quantifying rushing as half of the median time individuals spent to complete a soft launch of the survey (*N* = 50). To screen for bots, the researchers removed data when nonsensical answers were entered for both of two open-ended questions immediately following the messages. The protocol was approved by the University of Utah Institutional Review Board.

Data from 469 participants residing in the United States were included in the study. Qualtrics works to produce a sample that reflects the United States population in terms of sex, race and age. Participants had an average age of 43.06 years (SD = 16.86, range 18–80), and approximately half identified as female (49.7%, *N* = 233). Participants identified as white (81.7%, *N* = 383), Hispanic or Latina/o (14.3%, *N* = 67), Black or African American (10.9%, *N* = 51), Asian or Pacific Islander (5.3%; *N* = 25), American Indian or Alaskan Native (3.2%, *N* = 15) or an unlisted race (3.4%, *N* = 16). A little more than half of participants had a high school education or less (54.6%, *N* = 256).

### Stimuli

At the end of May 2020, news outlets had reported that the CDC had changed their position about the transmission of COVID-19 via surfaces.^38^ The CDC^39^ responded, claiming they had always communicated that surface transmission risk was low, and the supposed change in position was based off an edit made to their website to clarify their position, not to change it. Examination of statements made by the CDC in previous months supports the CDC’s claims.

The misinformation message was modeled after news stories covering the supposed change in position.^38^ The message highlighted that the CDC had reported finding COVID-19 in cruise ship rooms 17 days after passengers had left the ship—citing this as a reason the public was so concerned about surface transmission. The message then showed reporting from NBC’s Today and the Washington Post about the CDC’s change in position on COVID-19 surface transmission, indicating that the CDC’s ‘new’ guidelines were that COVID-19 did not spread easily on surfaces. The message ended with a quote from Columbia University virologist Angela L. Rasmussen, expressing concern over the CDC’s lack of clear communication with the public. All stimuli can be viewed via this Open Science Framework link: https://osf.io/ex4p2/?view_only=a0800569675a47dfbc21c1ec4a3a51de.

The didactic corrective highlighted refutations from CDC spokesperson Ben Haynes, including an explanation about why the virus material found on the cruise ship was probably not infectious. It compared the content on the CDC website from before and after the edit, demonstrating that the claims about surface transmission are the same and that the primary change was the header which originally read ‘Spread from contact with contaminated surfaces or objects’ and was changed to ‘The virus may be spread in other ways.’

The narrative corrective told the story of Jamie, who had been diligently cleaning packages and her groceries to protect against COVID-19 (despite her mother telling her it was unnecessary). The story about COVID-19 being found on a cruise ship after 17 days spiked her anxiety. When she subsequently read news stories about the CDC changing their position about surface transmission, she was frustrated and vented about it to her friends and on social media. A friend then points her to information demonstrating that the CDC did not, in fact, change their position. Like the didactic corrective, the narrative shows the content on the CDC website from before and after the edit and explains why the virus material on the cruise ship was likely not infectious. The story ends on a positive note with Jamie calling her mom to laugh and tell her that she was right about not needing to clean her packages.

### Measures

#### Misinformation belief

Misinformation belief about the CDC was captured using the item ‘The CDC recently changed positions about COVID and how long it lasts on surfaces.’ Participants responded on a scale from 1 (‘strongly disagree’) to 7 (‘strongly agree’) (*M* = 4.82, SD = 1.78). The item was embedded in a list of other statements about the CDC and COVID-19. See Table 1 for correlations between study variables.

**Table 1:** Correlation matrix

Variable	1	2	3
1. Misinformation belief	—		
2. Perceived CDC competence	−0.02	—	
3. Mask wearing intention	0.11^*^	0.31^**^	—
4. Relief	0.03	0.32^**^	0.16^**^

^*^ *P* ≤ 0.05.

^**^ *P* ≤ 0.001.

#### Perceived CDC competence

Perceived CDC competence was measured using the competence subscale of McCroskey’s source credibility scale.^40^ The four items were on a five-point scale ranging from 1 (‘strongly disagree’) to 5 (‘strongly agree’) and asked if the CDC is ‘intelligent’, ‘expert’, ‘competent’ and ‘trained’ (α = 0.89, *M* = 3.74, SD = 0.83).

#### Mask wearing intention

Participants were asked how likely they were on a scale from 1 (‘extremely unlikely’) to 7 (‘extremely likely’) to ‘wear a mask in public’ to slow the spread of COVID-19 (*M* = 5.69, SD = 1.91).

#### Relief

Relief was measured using four items. Participants indicated from 1 (‘none of this emotion’) to 7 (‘a great deal of this emotion’) how strongly the message made them feel ‘relieved’, ‘reassured’, ‘release’ and ‘consoled’ (α = 0.93, *M* = 3.93, SD = 1.60).

#### Political ideology

For political ideology, participants were asked ‘how would you describe your own political viewpoint?’ Responses included very liberal (*N* = 50, 10.7%), liberal (*N* = 89, 19%), moderate (*N* = 189, 40.3%), conservative (*N* = 80, 17.1%) and very conservative (*N* = 61, 13%).

## Results

### Hypotheses 1–3: direct effects of the narrative corrective

To test hypotheses 1–3, a series of ANOVAs were conducted using a dummy coded study condition variable (0 = no-correction control, 1 = didactic corrective, 2 = narrative corrective). ANOVA indicated a significant difference in misinformation belief by study condition, *F*(2, 466) = 34.90, *P* ≤ 0.001. Pairwise comparisons with least significant difference adjustments revealed that the narrative corrective resulted in a significantly lower misinformation belief (*M* = 4.02, SE = 0.13) than the control (*M* = 5.60, SE = 0.14, *r* = 0.43) and the didactic corrective (*M* = 4.86, SE = 0.13, *r* = 0.23), providing support for H1. ANOVA identified no difference between the study conditions on perceived CDC competence, *F*(2, 466) = 0.32, *P* = 0.73, or mask wearing intention, *F*(2, 466) = 0.69, *P* = 0.50. Therefore, neither H2 nor H3 were supported. See Table 2 for means and standard deviations by study condition.

**Table 2:** Means and standard deviations by study condition

Variable	Narrative corrective	Didactic corrective	No-correction control
Misinformation belief	**4.02 (1.82)**	**4.86 (1.68)**	**5.60 (1.47)**
Perceived CDC competence	3.78 (0.82)	3.71 (0.86)	3.75 (0.80)
Mask wearing intention	5.76 (1.83)	5.75 (1.89)	5.54 (2.02)
Relief	**4.24 (1.46)**	**3.65 (1.65)**	**3.92 (1.65)**

Notes*.* Mean (Standard Deviation). Bolded groups are significantly different (*P* < 0.05).

### Hypotheses 4–6: indirect effects via relief

To test hypotheses 4–6, a series of simple mediation analyses were conducted using PROCESS model 4 in SPSS, with separate analyses for each outcome. These analyses compared the effects of the narrative and didactic corrective. An indirect effect is present if the 95% confidence interval does not include zero.^41^

The narrative corrective had a positive, indirect effect through relief on perceived CDC competence (effect = 0.11, Boot SE = 0.04, 95% Boot CI: 0.0448, 0.1862) and mask wearing intention (effect = 0.15, Boot SE = 0.06, 95% Boot CI: 0.0540, 0.2714), but not on misinformation belief (effect = 0.07, Boot SE = 0.05, 95% Boot CI: −0.0154, 0.1638). Exposure to the narrative corrective resulted in greater relief (coefficient = 0.59, SE = 0.17, *t* = 3.36, *P* ≤ 0.001). When relief was higher, perceived CDC competence (coefficient = 0.19, SE = 0.03, *t* = 6.60, *P* ≤ 0.001) and mask wearing intention (coefficient = 0.25, SE = 0.07, *t* = 3.83, *P* ≤ 0.001) were higher. Therefore, H5 and H6, but not H4, were supported.

### Research question: differences by political ideology

We tested the research question using PROCESS model 59. PROCESS examined the significance of the associations at three levels of the five-point moderator including liberal (2), moderate (3) and conservative (4). Two differences were identified. First, the narrative corrective had a positive indirect effect on perceived CDC competence via relief for conservatives (effect = 0.14, SE = 0.05, 95% Boot CI: 0.0431, 0.2508) and moderates (effect = 0.09, SE = 0.03, 95% Boot CI: 0.0316, 0.1615) but not for liberals (effect = 0.05, SE = 0.04, 95% Boot CI: −0.0114, 0.1319). Similarly, the narrative corrective had a positive indirect effect on mask wearing intention via relief for conservatives (effect = 0.24, SE = 0.09, 95% Boot CI: 0.0678, 0.4312) and moderates (effect = 0.11, SE = 0.11, 95% Boot CI: 0.0287, 0.2006) but not for liberals (effect = 0.02, SE = 0.03, 95% Boot CI: −0.0493, 0.0914).

## Discussion

### Main finding of this study

The current study tested the effectiveness of narrative, compared with didactic, correctives of misinformation about the CDC’s COVID-19 messaging. The narrative corrective directly reduced misinformation belief compared with a didactic corrective and a no-correction control. For political moderates and conservatives, the narrative corrective also had a positive indirect effect on perceived CDC competence and mask wearing intentions through relief.

### What is already known on this topic

Narratives can be more persuasive than non-narratives because they generate greater emotion, are transporting and reduce reactance.^18–20^^,^^26–28^ However, comparisons between narrative and didactic correctives have produced mixed findings.^25^ Sangalang *et al*.^16^ suggest that including emotional endings will strengthen narrative correctives, supporting this supposition for negative emotional endings. Emotional flow research has found that starting a narrative with negative feeling and flowing into a positive ending is highly persuasive in non-corrective contexts.^30–32^ Misinformation about politics and outbreaks can be particularly difficult to correct,^23^^,^^24^ possibly necessitating a narrative strategy.

### What this study adds

The current study demonstrated that narrative messaging can be an effective strategy for correcting misinformation about a public health organization, specifically the CDC. The narrative outperformed the didactic corrective in mitigating the continued influence of misinformation. This finding ran counter to Ecker *et al*.’s work,^22^ which found no difference between narrative and non-narrative correctives. Ecker *et al*.’s^22^ research had excellent experimental fidelity—the narratives were very similar to the non-narratives, isolating narrativity. Conversely, the narrative in the current study included character background, feelings and internal thought processes. These details can give narratives greater fluency, meaning they are easier for audiences to process and comprehend.^42^^,^^43^ Fluency is a key factor in the success of correctives and could explain why the narrative in this study performed better than the didactic corrective.^12^ Future studies should test fluency as a mechanism and compare the effectiveness of narratives with or without these character details. Practically, our findings suggest that public health organizations should include narratives with positive emotion endings when addressing politically charged misinformation.

The study established that relief serves as a mechanism through which narrative correctives address affective belief echoes. The persuasiveness of the positive emotional ending is supported by emotional flow research.^30–32^ When designing corrective messaging, communicators should determine the most effective discrete emotion(s) to target for the context. Self-transcendent emotions (e.g. elevation, gratitude) could also be beneficial.^32^^,^^44^

The study also highlighted how relief can reduce affective belief echoes specifically for counter-attitudinal audiences. Relief was influential for conservatives and moderates. Corrective messaging is more likely to backfire for conservatives, compared with liberals, in some contexts.^13^ Narratives can reduce counterarguing, anger and other negative message responses compared with didactic messaging.^26^ Utilizing a narrative could therefore diminish the possible backfire effect of corrective messaging for conservatives. When narratives generate positive emotion, they can create an openness for considering diverse perspectives and opinions.^45^ Alternatively, liberals have higher acceptance of COVID-19 information and greater trust in the CDC and, therefore, may be more likely to accept corrective information regardless of its form.^36^^,^^46^^,^^47^

### Limitations of this study

The current study had several limitations that should be taken into account when interpreting the results. First, the source of the correction was the study itself.^48^ While the correction included references to the CDC’s corrective message, it did not frame the correction as coming from the CDC. Future research should assess if narrative correctives are considered effective and professional when coming from a source such as the CDC. Second, the study did not measure emotional reaction to the misinformation prior to the corrective. It is possible that the narrative corrective was more effective for audiences that experienced high levels of anger or anxiety related to the misinformation. Finally, the study did not include a condition in which misinformation was not presented, which could have served as a baseline for perceived CDC competence.

## Conclusion

Misinformation about public health organizations could damage the public’s confidence in those organizations and minimize adherence to health guidelines. During a health crisis, such as the COVID-19 pandemic, it is imperative to identify effective means of correcting such misinformation. The current study demonstrates the effectiveness of narratives with positive emotion endings for correcting misinformation about the CDC. The narrative indirectly affected the perceptions of the CDC’s competence and mask wearing intentions via relief for participants identifying as conservatives and moderates. The narrative did not have an indirect effect on those identifying as liberals, potentially because these individuals already had higher confidence in the CDC and intentions to wear a mask.

## Conflict of interest

The authors report that there are no competing interests to declare.

## Funding

This work was supported by the National Cancer Institute at the National Institutes of Health (grant numbers 1DP2EB022360–01, 3P30CA042014-29S7).

## Data availability

The data underlying this article cannot be shared publicly due to privacy reasons. Specifically, participants did not consent to having their data shared in a public repository. The data will be shared on reasonable request to the corresponding author.

## Notes

**Helen M. Lillie,** Assistant Professor

**Chelsea L. Ratcliff,** Assistant Professor

**Andy J. King,** Associate Professor

**Manusheela Pokharel,** Assistant Professor

**Jakob D. Jensen,** Professor
